# *Staphylococcus aureus* Endocarditis with Multivalvular Involvement Secondary to an Atrial Septal Defect

**DOI:** 10.1155/2016/3793968

**Published:** 2016-02-16

**Authors:** Vistasp Jimmy Daruwalla, Jahnavi Sagi, Hassan Tahir, Srikanth Penumetsa

**Affiliations:** Conemaugh Memorial Medical Center, Temple University, 1086 Franklin Street, Johnstown, PA 15905, USA

## Abstract

Infective endocarditis is usually diagnosed using modified Duke's criteria. Our patient had a subacute presentation and a low suspicion for endocarditis during admission, unfortunately leading to her death. Despite advances in diagnostic and therapeutic measures including antibiotic therapy and surgical techniques, morbidity and mortality with staphylococcal infective endocarditis remain high. Hence, we stress the significance of having a low threshold for TEE in patients with multisystem involvement due to* Staphylococcus aureus* that have evidence of persistent infection despite antibiotic treatment, even if the suspicion for endocarditis is low based on Duke's criteria. TEE substantially improves the sensitivity of diagnosis but may not be readily available in many medical centers. Presence of an ASD has been noted to have increased the risk of left sided endocarditis even with conditions that predispose to right sided endocarditis, particularly in patients with hemodialysis and diabetes as morbid risk factors.

## 1. Introduction

Infective endocarditis is a serious condition with a high mortality of 15 to 20%. Early diagnosis based on modified Duke's criteria involving a combination of clinical, microbiological, and imaging information is important [1]. Patients on hemodialysis are at increased risk of bacterial infections, particularly* Staphylococcus aureus*. Infective endocarditis can be a complication in up to 25% of these cases [2]. We report a case of culture negative, subacute infective endocarditis in a patient on hemodialysis, who also had an atrial septal defect (ASD), complicating the presentation.

## 2. Case Presentation

A 56-year-old female presented with a history of few days of generalized weakness and subjective fever. Her past medical history was significant for hypertension, type 2 diabetes mellitus, depression, and end-stage renal disease on hemodialysis via left AV fistula. Patient also complained of some urinary discomfort, nausea, and mild generalized abdominal pain but denied any chest pain or shortness of breath. She was afebrile with stable vital signs. Physical examination was significant for a widely split S2 and mild left costovertebral tenderness. Laboratory findings showed hemoglobin (Hb) of 8.5 gm/dL, WBC count of 24,000/cumm, and abnormal urine analysis, with +2 bacteria and significant proteinuria. A presumptive diagnosis of urinary tract infection was made and she was started on ceftriaxone for antibiotic coverage while awaiting urine and blood culture results.

Over the next 4 days, she became hypotensive and continued to have generalized fatigue with blood cultures negative for any growth. On day 5 there was pus drainage from the AV fistula access site during her dialysis. CT scan of her left upper extremity showed a small abscess with associated skin thickening and a poorly defined fluid collection at the site of her AV fistula. Surgical drainage was performed and pus culture subsequently grew rare colonies of* Staphylococcus aureus*. Patient was started on vancomycin and gentamycin for a broader empirical coverage. CT scan of her chest revealed bilateral pulmonary nodules consistent with an infectious or inflammatory process, highly suggestive of multiple septic emboli.

**Figure 1:**
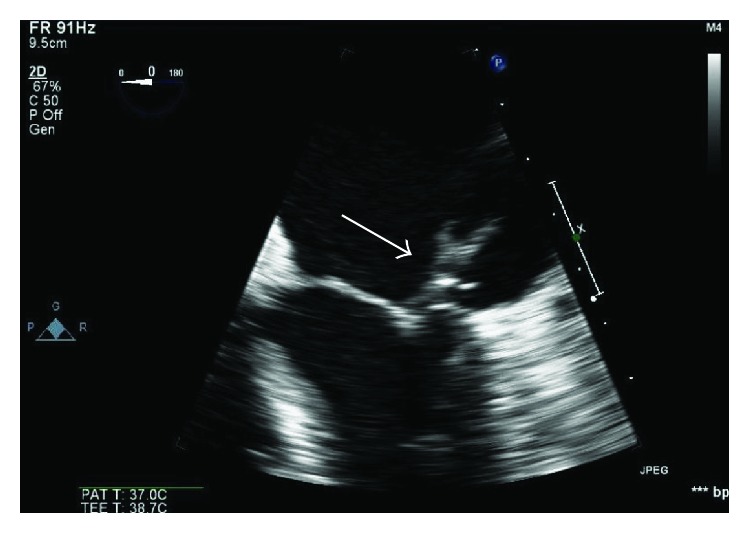
Mitral valve vegetation (arrow).

**Figure 2:**
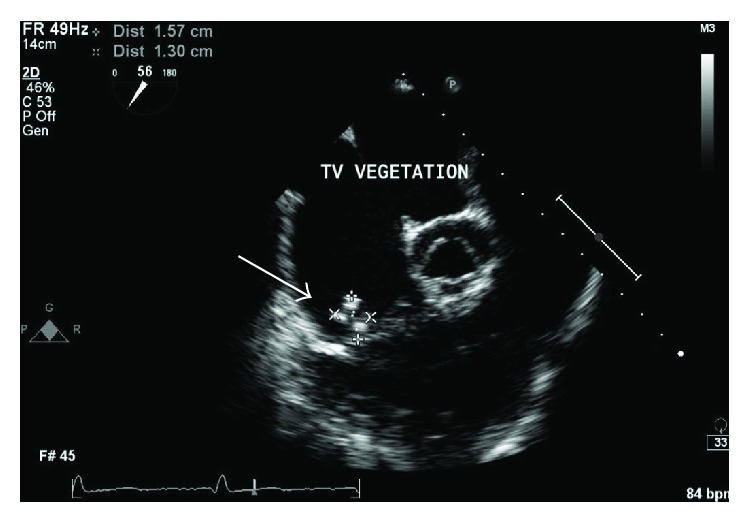
Tricuspid valve vegetation (arrow).

**Figure 3:**
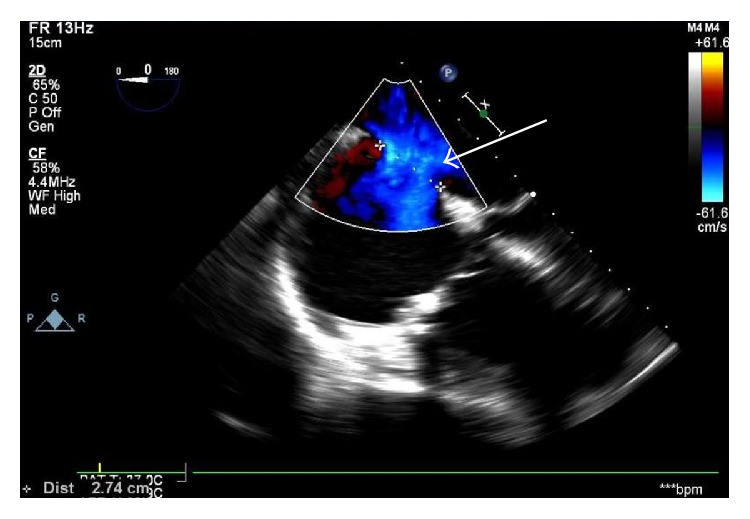
Atrial septal defect (arrow).

She continued to have persistent fatigue and markedly elevated ESR of over 140 mm/hr despite antibiotic treatment. Transesophageal echocardiogram (TEE) was performed, which showed a large vegetation (1.9 cm × 0.7 cm) located on the atrial aspect of the posterior mitral leaflet (Figure 1). The tricuspid valve demonstrated a fairly large (1.46 cm × 1.38 cm) vegetation, attached to the atrial aspect of the valve (Figure 2). Interatrial septum showed a large septum secundum type ASD measuring about 2.6 cm to 2.8 cm in diameter with color flow evidence of significant left to right shunt and intermittent right to left shunt across it (Figure 3). The Qp/Qs ratio was 1.56, indicating a significant shunt. A transthoracic echocardiogram 2 years ago showed normal structure of mitral and tricuspid valves with mild to moderate tricuspid regurgitation and moderate pulmonary hypertension. Cardiac catheterization showed severe disease of the left circumflex and right coronary artery. Patient underwent coronary artery bypass grafting along with closure of a large atrial septal defect with a bovine pericardium patch, mitral valve replacement with a tissue prosthesis, and tricuspid valve repair. Patient was subsequently transferred to the intensive care unit and was extubated the next day. Unfortunately the patient succumbed to sepsis and died.

## 3. Discussion

*Staphylococcus aureus* is a very virulent pathogen causing wide range of infections from benign soft tissue to lethal infective endocarditis, septic embolism, and toxic shock syndrome.* Staphylococcus* endocarditis is a critical illness with an overall mortality of 22–34%. The primary factor contributing to mortality is frequent delay in diagnosis and treatment due to the nonspecific nature of the symptoms [3].

The clinical presentation of this patient was intriguing as she had a low suspicion for endocarditis at admission, based on the modified Duke's criteria; she was afebrile and had multiple negative blood cultures. This is an unusual presentation for* Staphylococcus aureus*, which typically presents with acute endocarditis leading to significant damage of the valve. In our patient, infection at multiple sites, persistent symptoms, and markedly elevated ESR despite prompt antibiotic treatment subsequently raised the suspicion for endocarditis. It is likely that our patient had AV fistula site infection that spread to the tricuspid valve, which resulted in septic pulmonary emboli and seeding of the mitral valve across the large ASD.

## 4. Conclusion

Our case highlights a few important issues about* Staphylococcus aureus* infection in dialysis patients. Firstly, these patients may have an atypical presentation. Secondly applying modified Duke's criteria to determine the pretest probability for endocarditis, as a means to trigger an echocardiogram, may not be very sensitive in these patients; our patient did not have enough clinical suspicion for endocarditis initially, to trigger an echocardiogram. Despite advances in diagnostic and therapeutic measures, including antibiotic therapy and surgical techniques, morbidity and mortality with staphylococcal infective endocarditis remain high, with around 20% in hospital mortality and 27% at six months [4]. Hence, it is very reasonable to have a low threshold to perform an early TEE in patients with* Staphylococcus aureus* infection, who are at high risk for multisystem involvement.
